# Assessing the vulnerability of urban public health system based on a hybrid model

**DOI:** 10.3389/fpubh.2025.1576214

**Published:** 2025-05-21

**Authors:** Lingmei Fu, Zhirong Wang, Yutao Zhu, Benbu Liang, Ting Qian, Haiyun Ma

**Affiliations:** ^1^College of Emergency Management, Nanjing Tech University, Nanjing, Jiangsu, China; ^2^School of Management, Wuhan University of Technology, Wuhan, Hubei, China; ^3^School of Law, Humanities and Sociology, Wuhan University of Technology, Wuhan, Hubei, China

**Keywords:** urban public health system, vulnerability assessment, influential factor, weight determination, public health management

## Abstract

**Background:**

Public health emergencies pose direct threats to economic and social development. The vulnerability of urban public health system is a major cause of public health emergency outbreaks. It is essential to assess the vulnerability urban public health system.

**Materials and methods:**

To address the uncertainty inherent to the vulnerability assessment process, a novel hybrid model is proposed. Stage 1 involves the development of an indicator system, incorporating a comprehensive set of vulnerability factors identified through literature review and expert consultation. Stage 2 involves the calculation of indicator weights using the Bayesian best-worst method (BWM)—a novel probabilistic group decision-making approach that incorporates Bayesian statistics with the traditional BWM. Stage 3 involves the determination of vulnerability levels using a cloud model. The cloud model can combine the randomness and fuzziness of assessment to deal with uncertainty. The model is applied to assess the vulnerability of Shanghai's public health system. Moreover, a sensitivity analysis was conducted to validate the effectiveness and robustness of the model.

**Results:**

A total of 18 factors were identified as affecting the vulnerability of the urban public health system. The most significant among them are “poor coordination and cooperation among various personnel,” “insufficient information assurance,” “low public awareness,” and “low competency among staff in relevant departments and institutions.”

**Conclusion:**

The proposed hybrid model is both effective and robust. This study contributes to reducing the vulnerability of urban public health systems, thereby enhancing public health risk management in urban settings.

## 1 Introduction

The occurrence of public health emergencies, such as SARS and the Ebola outbreak in West Africa, not only posed direct threats to public health and safety but also adversely affected regional economic and social development (1). Therefore, preventing and controlling public health emergency is an essential prerequisite for achieving sustainable and high-quality urban development. The more vulnerable a public health system is, the greater the risk of such emergencies. The outbreak of public health emergencies exposes the vulnerability of urban public health system (U-PHS), which is a complex system (2). Vulnerability is a fundamental characteristic of complex systems (3), and it typically becomes apparent only when the complex system is subjected to certain disturbances. The degree of this vulnerability determines both the likelihood and severity of potential damage to the system. Consequently, reducing the vulnerability of U-PHS is crucial to promoting sustainable economic and social development.

Vulnerability and hazard are concomitant (4). The concept of vulnerability stems from research on natural disasters. In recent years, the concept of vulnerability has been widely used in many disciplines, such as health vulnerability (5) and socioeconomic vulnerability (6). The vulnerability of the public health system lies in its normal operation and function, which can be difficult to maintain due to high sensitivity, low adaptability, and limited recoverability when subjected to internal and external disturbances. U-PHS vulnerability can reflect whether the development of U-PHS is healthy and stable. U-PHS vulnerability assessment is the process of analyzing the capacity of U-PHS to withstand and respond to various public health risk factors. Vulnerability assessment is a crucial aspect of risk management and decision-making. It aids in predicting and assessing potential public health emergency risks, thereby providing decision support for the prevention and control of public health emergencies. Thus, assessing the vulnerability of U-PHS is of great significance.

Given the recurring outbreaks of public health emergencies, scholars have carried out some studies on U-PHS vulnerability. Based on the seasonally adjusted Poisson regression model, time-series regression analysis, and episode analysis, Hajat et al. concluded that public health is more vulnerable in moderately cold weather, and vulnerable groups differ depending on the type of wintertime weather conditions they experience (7). Wu et al. identified eight indicators for assessing health vulnerability associated with heat wave exposure in terms of exposure, sensitivity, and adaptability. They adopted a hesitant analytic hierarchy process to determine the level of health vulnerability (8). Zong et al. determined indicator weights based on directional interaction analysis among indicators. A weighted technique for the order of preference by similarity to the ideal solution method was applied to assess heat-related health vulnerability (5).

Some scholars have studied health vulnerability in relation to meteorological hazards. Lowe et al. further investigated the relationship between public physical health vulnerability before, during, and after the flood. They found that women, the older adult, and children are more vulnerable to physical health issues than other groups during floods. After a flood, the health vulnerability of individuals over 65 years and men is greater than that of other groups (9). Ebi and Bowen pointed out that the impact of drought on health is mostly indirect. Drought can affect health in multiple ways, notably through threats to water security and food availability (10). Lee et al. concluded that diseases such as diarrhea, water-borne diseases, the availability of hospital beds, and the number of physicians are all related to health vulnerability. Using equal-weight and principal-component methods, Lee et al. predicted social and health vulnerability to floods in Bangladesh (11). Zhong et al. established the indicator system of health vulnerability and adaptation through a literature review and factor analysis. The indicator system was established from three aspects: exposure, sensitivity, and adaptability. Based on the results of the analysis using a quantile regression model, they concluded that the impacts of the key components on the risks of waterborne diseases post-flood are heterogeneous (12). Fan et al. studied health vulnerability from an economic perspective. They constructed a fixed-effect panel data regression model that integrates the three main factors of public health, economic growth, and urbanization (13). Cui et al. evaluated public health emergency vulnerability in the Guangdong–Hong Kong–Macao Greater Bay Area based on the entropy method. They established an indicator system from three aspects: sensitivity, coping capacity, and collaborative governance (14).

The spread of major infectious disease epidemics has a strong adverse influence on the world. There are some studies on vulnerability to major infectious disease epidemics. Mishra et al. constructed a major infectious disease vulnerability index based on the analytic hierarchy process (AHP). They found that population density has a significant impact on the vulnerability index (15). Shadeed and Alawna found that factors such as population, population density, the older adult population, accommodation and food service activities, school students, chronic diseases, hospital beds, health insurance, and pharmacies influence public vulnerability to infectious disease. Based on these eight indicators, the geographic information system, in combination with AHP, was used to estimate the vulnerability index (16).

Researchers have investigated urban public health vulnerability from various perspectives, such as weather (5, 7), meteorological hazards (9–12), and economic factors (13). Three conclusions can be drawn from this literature analysis. First, existing studies have focused on the assessment of health vulnerability, with fewer studies focusing on the assessment of public health vulnerability. Obviously, health vulnerability is different from public health vulnerability (17). Second, although scholars have not reached a consensus on the influencing factors of vulnerability, many think that demographic characteristics and the economy have an impact on vulnerability (8, 15). Many studies have not fully considered the factors influencing vulnerability. For example, Fan et al. (13), Lee et al. (11), Mishra et al. (15), Wu et al. (8), and Zhong et al. (12) failed to consider the impact of management dimension on vulnerability. However, machine, environment, management, and quality dimensions of other related personnel are also associated with the normal operation and functioning of U-PHS. Third, the majority of literature on the vulnerability assessment of U-PHS follows a logic of “establishing an indicator system—determining indicator weights—assessing the vulnerability” (11, 14–16). Many methods have been adopted to calculate indicator weights, such as AHP (15, 16), the principal component approach (11), and the entropy weight method (14). There are two types of uncertainties in assessing U-PHS vulnerability, namely randomness and fuzziness. The vulnerability is determined by a combination of influencing factors. Different influencing factors may not be at the same state level simultaneously (randomness). At the same time, the vulnerability level of the influencing factor may be between two vulnerability levels (fuzziness). However, the methods used in the majority of studies did not deal with randomness and fuzziness uncertainties (11, 12, 15, 16). Hence, it is still necessary to conduct a study on the assessment of U-PHS vulnerability. This study aimed to scientifically assess the vulnerability of U-PHS. Specifically, an indicator system and a model for assessing the vulnerability of U-PHS need to be developed.

To fill these existing study gaps, a novel hybrid model was developed for the vulnerability assessment of U-PHS. This study comprehensively identifies influencing factors from the perspective of system composition. An indicator system for evaluating U-PHS vulnerability is constructed from human, machine, environment, and management dimensions. Assessing U-PHS vulnerability involves a multi-indicator decision-making process. Multi-indicator decision-making methods are powerful tools (18). Best-worst method (BWM) is a novel and promising multi-indicator decision-making method (19, 20). Compared with AHP, BWM has many advantages: it needs fewer pairwise comparisons [BWM: 2*n –* 3, AHP: *n* ^*^ (*n –* 1) / 2], has higher consistency, lower complexity, and is easier to adopt (only uses integers in pairwise comparisons) (21). BWM has been widely used in many disciplines. For instance, it has been applied in assessing the age-friendliness of cities (22), evaluating resilience (23), and conducting risk analysis (24). Nevertheless, BWM cannot amalgamate the preferences of multiple decision-makers in group decision-making problems. In this regard, Mohammadi and Rezaei applied Bayesian statistics to BWM and proposed Bayesian BWM (B-BWM) (25). B-BWM is a probabilistic group decision-making model that can quickly aggregate expert opinions to determine the weights of indicators with a small amount of calculation. Thus, this study explores the application of B-BWM in the vulnerability assessment of U-PHS. B-BWM cannot deal with the randomness and fuzziness uncertainties in the process of assessing vulnerability, while the cloud model can assess these uncertainties (26). Although the cloud model cannot determine weight indicators, it has four advantages: it combines the randomness and fuzziness of assessment to deal with uncertainties; the assessment accuracy of qualitative problems is high; it realizes the transformation between a qualitative concept and its quantitative instantiations, avoiding the distortion and loss of information. For a large number of fuzzy concepts in social and natural sciences, the expected curve of the cloud approximates a normal or semi-normal distribution. Therefore, the universality and scientific rationality of the cloud model have led to its application in many fields. Too scientifically assess the vulnerability of U-PHS, this study attempts to integrate B-BWM and the cloud model.

There are two innovations in this study. The first innovation is to comprehensively construct an indicator system for assessing U-PHS vulnerability from human, machine, environment, and management dimensions. This is the first study to attempt an assessment of the vulnerability of U-PHS. The second innovation is to develop a novel hybrid model for assessing U-PHS vulnerability that can take the uncertainties in the process of assessing vulnerability into account. Integrating a literature review and expert opinion, B-BWM, and the cloud model, the hybrid model is developed. Assessing U-PHS vulnerability is a multiple-criteria decision-making problem. Hence, this study introduces the B-BWM and cloud models into the vulnerability assessment of U-PHS, which enriches the research ideas. The paper provides a method for assessing the vulnerability of U-PHS, which is conducive to the scientific analysis of the vulnerability level of U-PHS and thus provides useful references for guaranteeing the healthy operation of U-PHS and preventing public health emergencies.

## 2 Methodology

A hybrid model consisting of three stages was proposed. Stage 1 constructs the indicator system for assessing the vulnerability of U-PHS. Stage 2 determines each indicator weight based on B-BWM. Stage 3 develops a comprehensive assessment of the cloud model based on the cloud model and determines the vulnerability level. This section mainly presents B-BWM and the cloud model.

### 2.1 Stage 1: construct an indicator system based on literature review and expert opinion

From the perspective of system composition, the indicator system was constructed based on four dimensions: human, machine, environment, and management. Indicators were determined based on a literature review and expert consultation.

### 2.2 Stage 2: calculate the weights of indicators adopting B-BWM

In B-BWM, both inputs and outputs are modeled as probability distributions. B-BWM treats the expert's assessment information as statistical samples and estimates the probability based on the statistical samples to determine the weight. B-BWM has been adopted in some disciplines (27).

Assume that there are *N* indicators and *K* experts, with the set of indicators being *A* = {*A*_1_, *A*_2,…_, *A*_*n*_}. B-BWM consists of four stages.

Step 1: Every expert is invited to identify the best indicator and the worst indicator.

Step 2: Determine Best-to-Others vector ($A_{B}^{k}$) and Others-to-Worst vector ($A_{W}^{k}$).

The expert uses a scale of 1–9 (1 means equally important, 9 means extremely more important) to determine the preference between indicators. In the opinion of expert *k*, $A_{B}^{k}$ is the best-to-Others vector.


(1) $$\begin{array}{l} A_{B}^{k}=\left(a_{B1}^{k},a_{B2}^{k},...,\;a_{Bn}^{k}\right),\;k\text{=1, 2,}...,K \end{array}$$

where $a_{Bj}^{k}$ indicates the preference of indicator *A*_*B*_ over indicator *A*_*j*_ in the opinion of expert *k*. Obviously, $a_{BB}^{k}\text{=}1,\;k\text{=1, 2,}...\text{,}K$.

Similarly, the Others-to-Worst vector $A_{W}^{k}$ is expressed as follows:


(2) $$\begin{array}{l} A_{W}^{k}={\left(a_{1W}^{k},\;a_{2W}^{k},\;...,\;a_{nW}^{k}\right)}^{T},k\text{=1, 2,}...\text{,}K \end{array}$$

It is clear that $a_{WW}^{k}=1,\;k\text{=1, 2,}...\text{,}K$.

Step 3: Calculate the optimal weight w^*^.

The indicator can be mapped into a random event, and the weight of the indicator can be mapped into the probability of occurrence. Therefore, it is reasonable to integrate probability distribution with BWM. Moreover, the calculation of indicator weights is transformed into the calculation of probability distribution.

In probability models, all inputs and outputs are modeled as probability distributions. Vectors $A_{B}^{k}$ and $A_{W}^{k}$ are the input vectors to the B-BWM. The elements in vectors $A_{B}^{k}$ and $A_{W}^{k}$ are integers. Therefore, multinomial distribution is able to model vectors $A_{B}^{k}$ and $A_{W}^{k}$. Assume that *w*^*k*^ is the weight vector under expert *k* opinion. *w*^*k*^ can be obtained based on Equations (3) and (4).


(3) $$\begin{array}{l} \left(A_{B}^{k}|w^{k}\right)\sim multinomial\left(1/w^{k}\right),\forall k=1,...,K \end{array}$$

(4) $$\begin{array}{l} \left(A_{W}^{k}|w^{k}\right)\sim multinomial\left(w^{k}\right),\forall k=1,...,K \end{array}$$

where multinomial is multinomial distribution.

In Bayesian inference, the Dirichlet distribution is used as the prior to the multinomial. It is scientific to use the Dirichlet distribution to analyze the weight vector. Assume that w^*^ is the optimal weight under group opinion.


(5) $$\begin{array}{l} \left(w^{k}|w^{*}\right)\sim Dir\left(\gamma *w^{*}\right),\;\forall k=1,\;2,\;...,K \end{array}$$

(6) $$\begin{array}{l} w^{*}\sim Dir\left(1\right) \end{array}$$

where *Dir* represents the Dirichlet distribution. γ is the concentration parameter that denotes the closeness between *w* and w^*^. Constrained by non-negativity, γ is modeled using a gamma distribution.


(7) $$\begin{array}{l} \gamma \sim gamma\left(a,b\right) \end{array}$$

where *a* and *b* represent the shape parameters of the gamma distribution. Generally, both *a* and *b* are set to 0.1 (28, 29). Based on the above equations, w^*^ can be computed by employing the Markov-chain Monte Carlo method.

Step 4: Analyze the confidence of w^*^.

The study employs credal ranking to test confidence for ranking. The confidence that indicator *A*_*i*_ is better than *A*_*j*_ is calculated by Equation (8).


(8) $$\begin{array}{l} CL\left(A_{i}>A_{i}\right)={\int I}_{\left(w_{i}^{*}>w_{j}^{*}\right)}P\left(w^{*}\right) \end{array}$$

In Equation (8), *CL* is the confidence level. The posterior distribution of w^*^ is *P*(w^*^). *I* is a conditional parameter. If $w_{i}^{*}>w_{j}^{*}$, *I* is 1, and zero otherwise. Having *Q* samples from the posterior distribution, the confidence is calculated as:


(9) $$\begin{array}{l} CL\left(A_{i}>A_{j}\right)=\frac{1}{Q}\sum_{Q=1}^{q}I\left(w_{i}^{*q}>w_{j}^{*q}\right) \end{array}$$

(10) $$\begin{array}{l} CL\left(A_{j}>A_{i}\right)=\frac{1}{Q}\sum_{Q=1}^{q}I\left(w_{j}^{*q}>w_{i}^{*q}\right) \end{array}$$

where w^*q^ is the *q-*th sample of w^*^from the samples. The higher the confidence level is to 1, the higher the reliability of weights is. Moreover, 0.5 is generally set as a threshold. When *CL*(*A*_*j*_ > *A*_*i*_) > 0.5, then indicator *A*_*j*_ is more important than *A*_*i*_. *CL*(*A*_*i*_ > *A*_*j*_) + *CL*(*A*_*j*_ > *A*_*i*_) = 1.

### 2.3 stage 3: determine the level of vulnerability employing the cloud model

To address uncertainties, the cloud model is used to effectively convert precise values (actual scores of indicators) into appropriate qualitative concepts (vulnerability level).

In the cloud model, the quantitative characteristics of the qualitative concept are represented by three parameters: expectation *Ex*, entropy *En*, and hyper entropy *He*. *Ex* is the expectation that the cloud drop belongs to a concept in the universe. *En* reflects the degree of uncertainty. The larger the *En* is, the more macroscopic the qualitative concept is and the wider the cloud map span is. *He* is the entropy of entropy, which is a measure of the uncertainty of entropy. It is determined by the randomness and fuzziness of entropy. The larger the *He* is, the greater the randomness of the certainty degree is, and the thicker the cloud is. The steps for establishing the assessment cloud model are as follows.

Step 1: Construct a benchmark for vulnerability assessment. Define the number of levels of vulnerability and the standard assessment cloud for each vulnerability level.

Step 2: Obtain each indicator's score and use the reverse cloud generator to calculate three parameters of the cloud model for each indicator.

Step 3: Calculate the comprehensive vulnerability assessment cloud for U-PHS. Based on the cloud-weighted arithmetic averaging operator algorithm, aggregate the cloud models of each indicator to obtain the comprehensive vulnerability assessment cloud for U-PHS.

Assume that there are *N* indicators. The three parameters of the cloud model for indicator *i* are *Ex*_*i*_, *En*_*i*_, and *He*_*i*_. Moreover, the weight of indicator *i* is *w*_*i*_. Equations (11)–(13) can be used to compute the three parameters (Ex^*^,En^*^, He^*^) of the comprehensive vulnerability assessment cloud model (30).


(11) $$\begin{array}{l} Ex^{\text{*}}\text{=}\overset{n}{\underset{i=1}{\sum }}w_{i}Ex_{i} \end{array}$$

(12) $$\begin{array}{l} En^{\text{*}}\text{=}\sqrt{\overset{n}{\underset{i=1}{\sum }}w_{i}En_{i}^{2}} \end{array}$$

(13) $$\begin{array}{l} He^{\text{*}}\text{=}\sqrt{\overset{n}{\underset{i=1}{\sum }}w_{i}He_{i}^{2}} \end{array}$$

Step 4: Determine the vulnerability level of U-PHS.

Assess the similarity between the comprehensive vulnerability cloud and each standard assessment cloud. The level of vulnerability is the linguistic term in which the standard assessment cloud with the largest similarity is located. Relevant preliminaries about cloud models and the calculation of the similarity between cloud models are presented in the Supplementary Appendix.

## 3 Results

Shanghai is a typical urban area with a large population, an advanced economy, convenient transportation, and has ties with other cities around the world. At the beginning of 2022, there were only sporadic domestic infectious disease cases in Shanghai. On March 28, Shanghai reported 96 new confirmed domestic infectious disease cases and 4,381 new asymptomatic domestic patients. On March 28th, Shanghai began to implement lockdown measures. Despite government interventions, the epidemic in Shanghai were not effectively controlled. The outbreak of the epidemic has brought serious consequences to Shanghai. This study uses Shanghai's public health system on 27 March 2022, as an example to carry out the practical application of the proposed model.

Similar to BWM, B-BWM does not require large samples. Considering that U-PHS involves multiple departments and multiple agents, we selected a middle-level manager of the government (Expert 1), a middle-level manager of the Shanghai Municipal Health Commission (Expert 2), and a middle-level manager of the Shanghai Municipal Center for Disease Control & Prevention (Expert 3). Three scholars who have more than 10 years of research experience were also invited (Experts 4, 5, and 6). These six experts were invited to conduct two questionnaire surveys about the importance of each indicator and the score of each indicator. The application of the proposed model is illustrated as follows.

### 3.1 Indicators determination

After initially determining the factors influencing vulnerability based on the literature review, the opinions of the above-mentioned six experts were consulted. Expert feedback was compiled and returned to the experts. And the indicators for assessing urban public health vulnerability were finally identified through online discussions among experts. U-PHS is a complex system composed of several elements, and the vulnerability of the elements affects the vulnerability of the system. In the opinion of the expert group, humans, machines, and the environment are the basic elements of U-PHS. Management is the key dimension to maintaining the normal operation of the system. Hence, the combined performance of human, machine, environment, and management components ensures the stable functioning of U-PHS. The vulnerability of U-PHS in human-machine-environment management is the root cause of the vulnerability of the system. A total of 18 sub-indicators from the four dimensions were used to construct an indicator system (see Table 1). The description of indicators is shown in the Supplementary Appendix.

**Table 1:** Weights of all indicators.

**Main-indicators**	**Sub-indicators**	**Local weights**	**Global weights**	**Ranking**
Human (A1)	Low public quality (A11)	0.2703	0.0924	3
	Low quality of medical and health institution staff (A12)	0.1778	0.0608	7
	Low quality of competent department and institution staff (A13)	0.2547	0.0871	4
	Low quality of public safety organization staff (except medical and health institution staff) (A14)	0.1230	0.0421	11
	Low quality of experts and medical researchers (A15)	0.0946	0.0324	15
	Low quality of media staff (A16)	0.0796	0.0272	16
Machine (A2)	Insufficient allocation and expansion capability of health resources (A21)	0.2953	0.0468	9
	Imperfect public utilities (A22)	0.2331	0.0370	13
	Insufficient allocation and scheduling capability of emergency resources (A23)	0.4715	0.0747	5
Environment (A3)	Low economic level (A31)	0.3780	0.0527	8
	Low science and technology level (A32)	0.3289	0.0458	10
	Poor environmental hygiene (A33)	0.1246	0.0174	18
	Occurrence of special background (A34)	0.1685	0.0235	17
Management (A4)	Poor coordination and cooperation among various personnel (A41)	0.3243	0.1168	1
	Insufficient information assurance (A42)	0.3019	0.1087	2
	Imperfect laws and regulations (A43)	0.1077	0.0388	12
	Insufficient emergency planning and drilling (A44)	0.1705	0.0614	6
	Insufficient training of relevant personnel (A45)	0.0955	0.0344	14

### 3.2 Weights calculation

The above six experts were invited to conduct pairwise comparisons between indicators. Taking the four main indicators as an example, Table 2 shows the pairwise comparison results of these four indicators under the six experts' opinions.

**Table 2:** Pairwise comparisons for main indicators.

**Experts**	**Best/Worst main-indicators**	**A1**	**A2**	**A3**	**A4**
Expert 1	Best (A1)	1	5	4	3
	Worst (A2)	5	1	3	4
Expert 2	Best (A1)	1	5	7	3
	Worst (A3)	7	3	1	5
Expert 3	Best (A4)	4	3	5	1
	Worst (A3)	3	4	1	5
Expert 4	Best (A1)	1	5	4	3
	Worst (A2)	5	1	2	4
Expert 5	Best (A4)	3	5	4	1
	Worst (A2)	4	1	3	5
Expert 6	Best (A4)	3	5	7	1
	Worst (A3)	5	3	1	7

**Figure 1:**
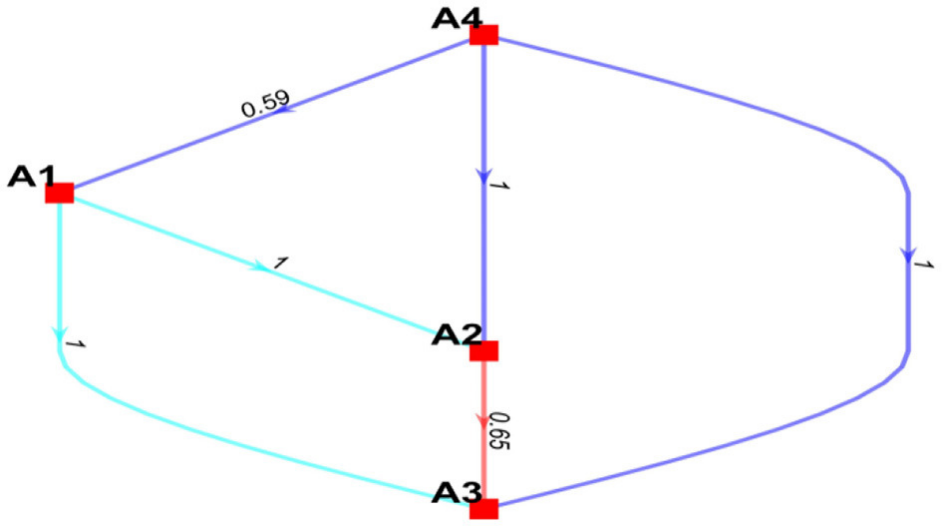
The visualization of the credal ranking for the main indicators.

Based on B-BWM, the weights of the four main indicators can be obtained using the data in Table 2 programmed in Matlab. The resulting weights for the A1, A2, A3, and A4 indicators based on group opinions are 0.3420, 0.1585, 0.1393, and 0.3602, respectively. Credit ranking can be used to assess the confidence of weights. The results are visualized (see Figure 1).

In Figure 1, edge $A\overset{conf}{\to }B$ means that indicator A is more preferred than indicator B with the confidence level *conf* . It can be seen that the management dimension is the most important dimension. The confidence levels of the management dimension are more important than the human dimension, machine dimension, and environment dimension, which are 0.59, 1, and 1, respectively. All confidence levels are >0.5. Consequently, the weights of these four indicators and their rankings were deemed reliable.

**Figure 2:**
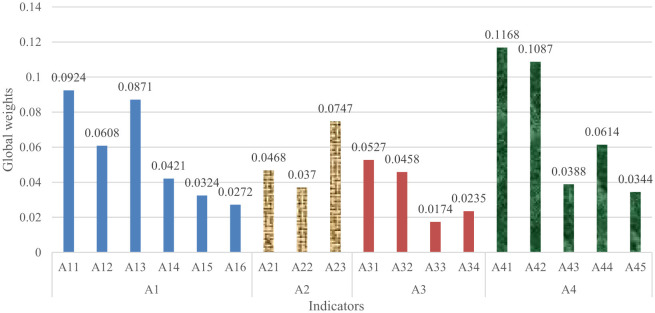
The weights of all indicators.

Based on the data from the pairwise comparisons carried out by the above six experts, the weights of sub-indicators and relevant confidence levels were obtained. By analyzing the relevant confidence levels, it can be concluded that the rankings of the importance of indicators are reliable. Figure 2 presents the weights of all indicators.

### 3.3 Vulnerability level analysis

According to cognitive research, humans can make correct judgments when five linguistic terms are in the linguistic term set. Hence, the level of U-PHS vulnerability was defined by the linguistic term set with a scale of 5 (very low, low, medium, high, and very high). The corresponding five standard assessment clouds are generated based on the golden ratio section method. By setting the *He* of the third standard assessment cloud to 0.1, the numerical characteristics of standard assessment clouds can be generated (see Table 3).

**Table 3:** Standard assessment clouds and their numerical characteristics.

**Linguistic terms**	**Standard assessment clouds**
Very low	(0, 1.0302, 0.2618)
Low	(3.09, 0.6367, 0.1618)
Medium	(5, 0.3935, 0.1)
High	(6.91, 0.6367, 0.1618)
Very high	(10, 1.0302, 0.2618)

After determining the standard assessment clouds of vulnerability assessment, the six experts mentioned above conducted a questionnaire survey about the score of each indicator. To ensure the accuracy of results, we collected data and information about the overall situation in Shanghai, such as the number of hospital beds per 1,000 people and the proportion of the population aged 60 years and older. Relevant data and information were organized into a Word document and distributed to each expert so that they could provide a reference for scoring. Each indicator was scored on a 0–10 scale. The higher the actual score of each indicator, the more vulnerable U-PHS is. The scores of indicators under six experts' opinions are shown in Table 4. The numerical characteristics of the cloud model for each indicator were calculated based on the reverse cloud generator algorithm.

**Table 4:** Data on the vulnerability assessment of Shanghai's public health system and numerical characteristics of relevant cloud models.

**Sub-indicators**	**Expert 1**	**Expert 2**	**Expert 3**	**Expert 4**	**Expert 5**	**Expert 6**	***Ex***	***En***	***He***
A11	5	6	5	4	5	7	5.3333	0.9748	0.3412
A12	6	6	5	5	4	6	5.1667	0.6963	0.2861
A13	6	6	5	6	4	7	5.6667	0.9748	0.3412
A14	6	5	6	5	4	5	5.1667	0.6963	0.2861
A15	4	4	5	5	3	6	4.5	1.0444	0.0958
A16	5	4	5	7	6	7	5.8333	1.4622	0.1692
A21	6	5	5	6	6	7	5.8333	0.6963	0.2861
A22	6	6	5	5	5	4	5.1667	0.6963	0.2861
A23	8	8	7	7	9	9	8	0.8355	0.3192
A31	1	1	2	1	1	2	1.1667	0.3481	0.2132
A32	3	4	4	2	3	3	3.1667	0.6963	0.2861
A33	7	7	6	6	6	5	6.1667	0.6963	0.2861
A34	3	3	4	4	5	5	3.5	0.8355	0.3192
A41	8	8	10	9	8	8	8.5	0.8355	0.0432
A42	8	8	8	9	8	8	8.1667	0.3481	0.2132
A43	5	6	7	7	7	7	6.5	0.8355	0.0432
A44	7	8	8	8	7	9	7.8333	0.6963	0.2861
A45	5	6	7	7	7	7	6.5	0.8355	0.0432

**Figure 3:**
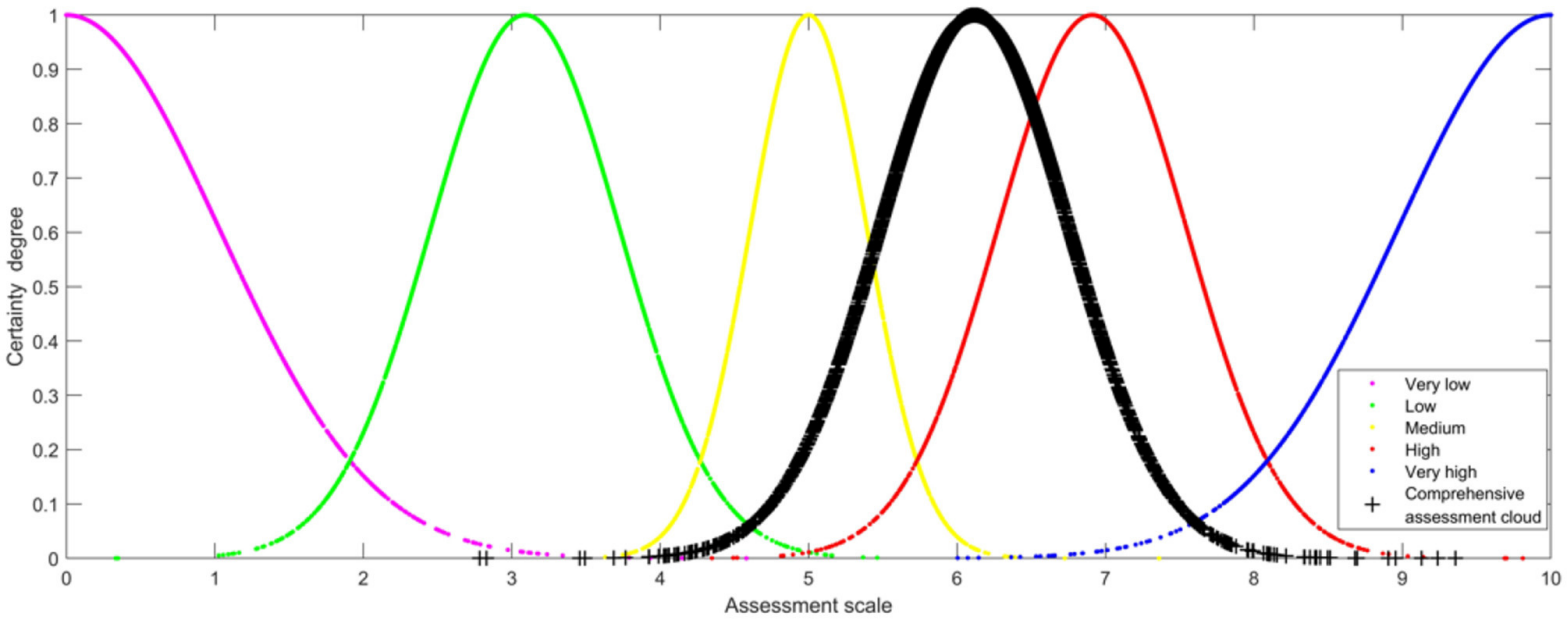
Comprehensive assessment cloud map of Shanghai's public health system vulnerability.

By aggregating the four cloud models, it can be concluded that the numerical characteristics of the comprehensive assessment cloud model are (6.1178, 0.6378, and 0.2552) (see Figure 3, *N* = 3,000).

The similarity between the comprehensive assessment of Shanghai's public health system vulnerability and the five standard assessment clouds was assessed. It can be found that the comprehensive assessment cloud of public health system vulnerability is most similar to the “High” standard assessment cloud. Therefore, Shanghai's public health system vulnerability on 27 March 2022 is high, which is in line with the actual situation.

## 4 Discussion

### 4.1 Analysis of indicator weight

Human dimension vulnerability (A1), with a weight of 0.3420, is the second important main indicator. Low public quality reflects low resilience to hazards, which is an important underlying cause of the outbreak and spread of public health emergencies. Hence, human vulnerability is a key determinant of the level of U-PHS vulnerability. Low public quality (A11), with a weight of 0.0924, is the critical sub-indicator of the human dimension, which echoes with the findings of some existing studies. Jaya and Folmer found that health conditions have a large impact on public self-immunity (31). Public health awareness and public health knowledge affect the public's ability to respond to emergencies (32). All of these factors can illustrate the importance of public quality to the vulnerability of U-PHS. The quality of competent department and institution staff, with a weight of 0.087, holds the second rank among the sub-indicators of the human dimension and the fourth rank among all sub-indicators. The quality of medical and health institution staff determines the level of medical treatment. Thus, “low quality of medical and health institution staff” (A12) is an important sub-indicator with a weight of 0.0608. The competent departments and institutions of public health are responsible for disease prevention and control, planning immunization, health surveillance, etc. These responsibilities have an important impact on the vulnerability of U-PHS. Compared with the impact of public health competent departments and institutions on U-PHS vulnerability, the quality of public safety organization staff, experts, and medical researchers has less impact on U-PHS vulnerability “low quality of media staff” (A16) has the least weight of 0.0272 in the human dimension, which may be the reason why there are few existing studies analyzing the impact of media on urban public health vulnerability.

The weight of machine dimension vulnerability (A2) is 0.1585. The importance of A2 ranks third among the main indicators. The more perfect the equipment/resource allocation is, the lower the vulnerability of U-PHS is and the higher the public health security is. The weight of “insufficient allocation and scheduling capability of emergency resources” (A23) is as high as 0.0747, ranking first in the sub-indicators of machine dimension and fifth in all sub-indicators. This may stem from the social nature of public health emergencies, in which the entire society. The social nature of public health emergencies leads to adverse effects in many aspects, including health impact, economic impact, and environmental impact. Thus, it is necessary to control the spread of public health emergencies in a timely manner. Emergency resources are the material guarantee for the handling and rescue of public health emergencies, and thus, great attention should be paid to the allocation and scheduling of emergency resources (33). Compared with “Imperfect public utilities” (A22), “Insufficient allocation and expansion capability of health resources” (A21) has a higher weight. Health resources are the basis for health departments providing the public with health services. Hence, health resources directly affect the treatment and quarantine of public health emergencies (34). This may contribute to the high weight of A21.

Environmental dimension vulnerability (A3), with a weight of 0.1393, holds the fourth rank among the main indicators. Ho et al. found that environment dimension vulnerability is significantly associated with health vulnerability (35). The weights of “Low economic level” (A31) and “Low science and technology level” (A32) are larger than the other two sub-indicators of A3. A31 and A32 rank eighth and tenth among all sub-indicators, respectively. This is consistent with the conclusion of existing studies (13). Economic development is beneficial to ensuring the operation of the public health system. Specifically, economic development positively affects the allocation of equipment/resources for medical care, housing, and health, which is conducive to maintaining the normal operation of U-PHS. Fan et al. found that economic growth is associated with lower health vulnerability. The collapse of the U-PHS will lead to the outbreak of public health emergencies, which in turn will bring economic losses (13). Compared to “poor environmental hygiene” (A33) and “special background” (A34), the weight of “low science and technology level” (A32) is larger. This may be due to the fact that the important role of scientific and technological support in the prevention and control of public health emergencies has become increasingly prominent. For example, in the early stages of the prevention and control of public health emergencies, science and technology plays an important role in the origin-tracing of the virus and analyzing the transmission and evolution mechanisms of the virus. After the outbreak of public health emergencies, scientific and technological forces in multiple fields can be gathered to control the spread of emergencies.

Management dimension vulnerability (A4) is the most critical main indicator. Its weight is as high as 0.3602. “Poor coordination and cooperation among various personnel” (A41) is found to be the most important sub-indicator of A4, followed by “Insufficient information assurance” (A42). The weights of A41 and A42 are 0.1168 and 0.1087, holding first and second rank among all sub-indicators, respectively. Coordination and cooperation among various personnel are not only prerequisites for the normal operation of U-PHS but are also conducive to the scientific and accurate implementation of prevention and control measures. In addition to the information interaction within U-PHS, there is also information interaction between U-PHS and the outside of the system. Information symmetry and high-efficiency information transmission are beneficial to the self-adaptive adjustment of U-PHS, reducing the vulnerability of the system. In order to reduce U-PHS vulnerability, the focus should be on improving coordination and cooperation among various personnel and providing information guarantees.

### 4.2 Validation of model effectiveness and robustness

Sensitivity analysis is an effective tool widely used by researchers to check the effectiveness and robustness of the model (21, 36, 37). Thus, this study analyzed the impact of sub-indicators' weight changes on the level of vulnerability. Following existing studies (36, 37), let the weight of each sub-indicator in Table 1 be a normalized scenario. Specifically, the weight of the highest-ranked indicator (A41) varied from 0.1 to 0.9, and other sub-indicator weights changed in proportion. In these nine scenarios, the similarity between the comprehensive vulnerability assessment cloud and the “High” standard assessment cloud is higher than the other four standard assessment clouds as a whole. Therefore, the conclusion that Shanghai's public health system was highly vulnerable is reliable. Thus, the effectiveness and robustness of the hybrid model in assessing U-PHS vulnerability have been confirmed. The proposed model accounts for uncertainties in the process of assessing vulnerability, and it can be used for vulnerability assessments in other fields.

### 4.3 Managerial implications

The hybrid model incorporating the cloud model realizes the scientific transformation from actual scores of indicators to Shanghai's public health system vulnerability on 27 March 2022. Shanghai's public health system vulnerability on 27 March 2022, is high. The vulnerability of the public health system and public exposure to a new kind of coronavirus caused the outbreak of an epidemic situation in Shanghai. Based on the results of this study, the management implications are as follows.

First, the public health management system should be improved. The government should continuously improve laws and regulations for the emergency management of public health emergencies. The public health emergency response planning system can be improved by building a health emergency team that encompasses the fields of virus detection, epidemiological investigation, medical treatment, community guidance, resource allocation, and so on. As public health products (e.g., vaccines, drugs, and diagnostic reagents) exhibit positive externalities typical of public goods, the government is expected to increase research and development funding for public health products. It is necessary for competent department and institution staff to improve crisis awareness, sense of responsibility and dedication, and learn new technologies and new theoretical knowledge. To improve the quality of medical staff, efforts should be made to strengthen the construction of medical disciplines and make the knowledge and skills of public health emergency prevention and control a compulsory course for medical staff. Particular attention ought to be paid to strengthening the team building of medical experts, especially the team building of experts on major infectious diseases to enhance the ability to treat diseases. Improving the guarantee system and incentive mechanism for public health-related agents is very conducive to enhancing the work enthusiasm of public health-related staff. Media personnel are able to reduce the vulnerability of U-PHS by shaping public opinion and spreading public health and emergency knowledge in a timely manner.

Second, efforts should be made to enhance public's ability to prevent and respond to diseases. On the one hand, individuals' self-protection and immunity should be improved. To reduce human-dimensional vulnerability, it is important to encourage the public to abandon harmful habits and adopt a healthy lifestyle. Effectively preventing and controlling chronic diseases is a practical approach to enhancing individual immunity. Additionally, improving public knowledge and awareness of health is crucial. Promoting lifelong learning in public health and leveraging both new media and traditional media for effective health education are vital strategies. The activities of new media personnel should be appropriately regulated. The use of new media plays an important role in popularizing public health knowledge. It is feasible to strengthen the role of traditional media in the dissemination of public health knowledge. In addition, improving public health service items is beneficial to comprehensively enhancing the public's ability to prevent diseases, thereby reducing the risk of public health emergencies.

Third, it strengthens coordination and cooperation among all agents and facilitates information transmission. Enhancing the awareness of coordination and cooperation among various agents is an essential prerequisite for promoting the realization of collaborative linkage between different agents. To ensure coordination among all agents and improve the efficiency of resource utilization, it is necessary to broaden the communication channel. It is necessary to develop effective messaging strategies in the public health field (38). Establishing information-sharing platforms and interactive information platforms with the help of big data, artificial intelligence, and cloud computing is essential to provide information guarantees for promoting coordination and cooperation among all agents. In addition, responding to public concerns and releasing authoritative information in a timely manner is needed. These actions are conducive to reducing the vulnerability of U-PHS in the four dimensions of human, machine, environment, and management.

## 5 Conclusion

Vulnerability effectively reflects a system's capacity to manage risk. Considering the uncertainty in the vulnerability assessment process, this study constructs a novel hybrid model for assessing the vulnerability of U-PHS. The model includes three stages. In stage 1, a literature review and expert consultation are needed to construct the index system, incorporating comprehensive vulnerability factors. In stage 2, a novel method based on probability distribution, namely B-BWM, is adopted to calculate indicator weights. B-BWM can scientifically aggregate the opinions of experts and then obtain aggregated weights. In stage 3, the cloud model is employed to analyze the vulnerability level of U-PHS. Based on theories of uncertainty, the cloud model realizes the scientific transformation from a precise quantitative value (actual scores of indicators) to a qualitative concept (vulnerability level). A sensitivity analysis was conducted to verify the effectiveness and robustness of the model.

The findings reveal that 18 factors affect U-PHS vulnerability. “Poor coordination and cooperation among various personnel,” “insufficient information assurance,” “low public quality,” and “low quality of competent department and institution staff” are the main factors leading to the vulnerability of U-PHS. The developed model is robust and reliable. Based on the identified key indicators, we propose targeted managerial implications: improve the public health management system, enhance public's ability to prevent and respond to diseases, strengthen the coordination and cooperation among all agents, and facilitate information transmission. The study provides decision support for reducing U-PHS vulnerability, thereby promoting urban public health risk management. Moreover, the hybrid model can be applied in other contexts, including different sectors and regions.

Although the effectiveness and robustness of the hybrid model for assessing U-PHS vulnerability have been verified, the study has a limitation. Only limited data on public health systems is available to protect privacy and ensure public information security. Similar to many studies, this study relied on expert opinions to determine the weight of each indicator, which introduces subjectivity into the process (36, 39). In future research, quantitative methods such as Bayesian networks and complex networks can be explored to assess vulnerability levels.

## Data Availability

Data generated in this study will be made available upon reasonable request.
