# Supplementary material for: Assessing the vulnerability of urban public health system based on a hybrid model
Source: Front Public Health. 2025 May 21;13:1576214. doi: 10.3389/fpubh.2025.1576214 (PMC12133845; doi:10.3389/fpubh.2025.1576214)
Supplement: Supplementary file 1 [file Data_Sheet_1.docx]

Supplementary Material

## 1. Description of indicators

**Human dimension vulnerability**. The vulnerability of each agent in urban public health system (U-PHS) leads to the vulnerability of the system. The public is the most direct and serious victims of public health emergency. Public quality includes public self-immunity, public health awareness, and public health knowledge. Public quality affects the vulnerability of public. Compared with adults, children and the elderly have lower self-immunity and are more sensitive to public health risk. Compared with other groups, people with underlying diseases are more likely to be infected with influenza, COVID-19 and other diseases. And people with underlying diseases have lower self-immunity, and are more vulnerable to negative health outcomes. Low level of public health awareness and public health knowledge is not conducive to the reduction of sensitivity and adaptability of U-PHS, which makes the system more vulnerable. The competent departments and institutions of public health system consist of health administrative departments at all levels, Centers for Disease Control and Prevention, health supervision and management institutions, etc. Quality of competent departments and institutions staffs inevitably affects the level vulnerability of public health system. Public safety organization includes ministry of public security, fire brigade, general customs administration, the food and drug administration, medical emergency center and so on. It should be noted that as mentioned earlier, medical and health institution are on the frontline defense in the prevention and control of the epidemic and medical treatment, thus this paper took “Low quality of medical and health institution staffs” as an indicator. And “Low quality of public safety organization staffs (except medical and health institution staffs)” was took as another indicator. During the prevention and control of public health emergency, experts can carry out science popularization, can stabilize public psychology and provide professional advice for effective prevention and control of emergency. Medical researchers play an essential role in rapid diagnosis, treatment method, vaccine research and development and so on. Media is the bridge of communication between government and public. Hence, the quality of experts, medical researchers, and media staffs has an impact on the vulnerability of U-PHS.

**Machine dimension vulnerability**. The definition of machine here is broad, including equipment, resource, etc. Insufficient allocation and expansion of health resources [1], imperfect public utilities, and insufficient allocation and scheduling of emergency resources have an influence on U-PHS vulnerability [2]. Health resource is the collective term of all kinds of production factors occupied or consumed in the process of providing health services. Health resources include medical institutions, medical beds, medical equipment, medical supplies, etc. It is worth noting that, in order to distinguish from human dimension, health resources here do not include medical staff. Perfect public utilities are conducive to improving the capacity of public health services, thereby improving the ability to deal with public health risks. For example, smooth traffic is benefit for the transportation of medical supplies. The construction of disease surveillance and early-warning system has a beneficial effect on timely preventing the outbreak of epidemic and blocking the spread of epidemic. Emergency resources refer to various resources (materials, funds, facilities, information, technology, etc.) needed in the emergency management system to effectively carry out emergency activities and ensure the normal operation of U-PHS. The less adequate the emergency resource is, the lower the emergency response capacity is, and the higher the vulnerability of U-PHS is.

**Environmental dimension vulnerability**. The environment of U-PHS mainly includes economic environment [3], scientific and technological environment, hygienic environment [1,3]. Among them, per capita GDP can refelect the current state of economic environment. Scientific and technological environment needs to be reflected from many aspects. For example, scientific and technological inputs, scientific and technological papers and patent output. Special background refers to the occurrence of natural disasters, accidents, and large events. The impact of special background such as drought and floods on public health is multifaceted, including psychological effect, physiological injury, disease, infections and so on [2].

**Management dimension vulnerability**. The lower the management level is, the more difficult it is for public health system to resist adverse disturbances, and the more difficult it is for public health system to adjust appropriately to changes in the environment. The coordination and cooperation among various personnel, information assurance are beneficial to the healthy operation of public health system. Promoting information symmetry and improving the efficiency of information transmission is the key to information assurance. Laws and regulations on public health have regulatory functions such as guidance, education, and enforcement. Laws and regulations can regulate the behavior of related agents and then reduce the vulnerability of the public health system. Emergency plan is a plan made in advance to ensure rapid, orderly and effective emergency response and rescue operations. The emergency drill of emergency plan is an important means to test, evaluate and maintain the emergency response and rescue capabilities. The training of relevant personnel facilitates U-PHS management.

## 2. Calculate the similarity between two cloud models

This study calculates the similarity between cloud models based on the shape similarity and distance similarity between two cloud models [4]. Let there are two cloud models, $D_{i}(Ex_{i},En_{i},He_{i})$, $D_{j}(Ex_{j},En_{j},He_{j})$. And the calculation steps of shape similarity and distance similarity between two cloud models are illustrated as follows.

Step 1: Calculate the shape similarity between two cloud models *S_1_*.

$S_{1}=\frac{min(\sqrt{En_{i}^{2},He_{i}^{2}},\sqrt{En_{j}^{2},He_{j}^{2}})}{max(\sqrt{En_{i}^{2},He_{i}^{2}},\sqrt{En_{j}^{2},He_{j}^{2}})}$ (1)

Step 2: Determine *k*,, and based on Equations (2)-(4).

$k=\frac{max(En_{i},En_{j})}{min(En_{i},En_{j})}$ (2)

(3)

(4)

Step 3: Calculate the distance similarity between two cloud models *S_2_*.

(5)

According to the values of *k* and , the values of *a*, *b*, and *c* can be obtained (please see Table 1).

**Table 1.** Related data on distance similarity between cloud models.

|  | *k* | *a* | *b* | *c* |
| --- | --- | --- | --- | --- |
| 0 | 1 | 1.42 | -0.3142 | 0.527 |
| 0.1 | 1.222 | 1.256 | -0.2038 | 0.4772 |
| 0.2 | 1.500 | 1.088 | -0.08523 | 0.4213 |
| 0.3 | 1.875 | 1.026 | -0.01631 | 0.393 |
| 0.4 | 2.333 | 1.007 | 0.02322 | 0.3859 |
| 0.5 | 3 | 1.002 | 0.048 | 0.3914 |
| 0.6 | 4 | 1.001 | 0.06496 | 0.4043 |
| 0.7 | 5.667 | 1.002 | 0.07731 | 0.4218 |
| 0.8 | 9 | 1.002 | 0.08654 | 0.4423 |
| 0.9 | 19 | 1.003 | 0.09325 | 0.4654 |
| 1 | 20 | 1.003 | 0.09759 | 0.4911 |

Step 4: Analyze the similarity between each standard assessment cloud model and comprehensive vulnerability assessment cloud based on both shape similarity and distance similarity. The level of vulnerability is the linguistic term in which the standard assessment cloud with the largest similarity is located.

## References

[1] Paul S. Vulnerability and the resilience against covid-19 in India[J]. J Asia Pac Econ, 2023, 28(4): 1373-1391. https://doi.org/10.1080/13547860.2021.1915545.

[2] Zhong S, Cheng Q, Huang CR, Wang Z. Establishment and validation of health vulnerability and adaptation indices under extreme weather events on the basis of the 2016 flood in Anhui province, China. Adv Clim Chang Res, 2021; 12(5): 649-659. https://doi.org/10.1016/j.accre.2021.07.002.

[3] Fan Y, Fang M, Zhang X, Yu Y. Will the economic growth benefit public health? Health vulnerability, urbanization and COVID-19 in the USA. Ann Regional Sci, 2023; 70(1): 81-99. https://doi.org/10.1007/s00168-021-01103-9.

[4] Wang J, Zhu J, Liu X. An integrated similarity measure method for normal cloud model based on shape and distance. Sys Eng Theor & Pra, 2017; 37(3): 742-751. https://doi.org/10.12011/1000-6788(2017)03-0742-10.

**
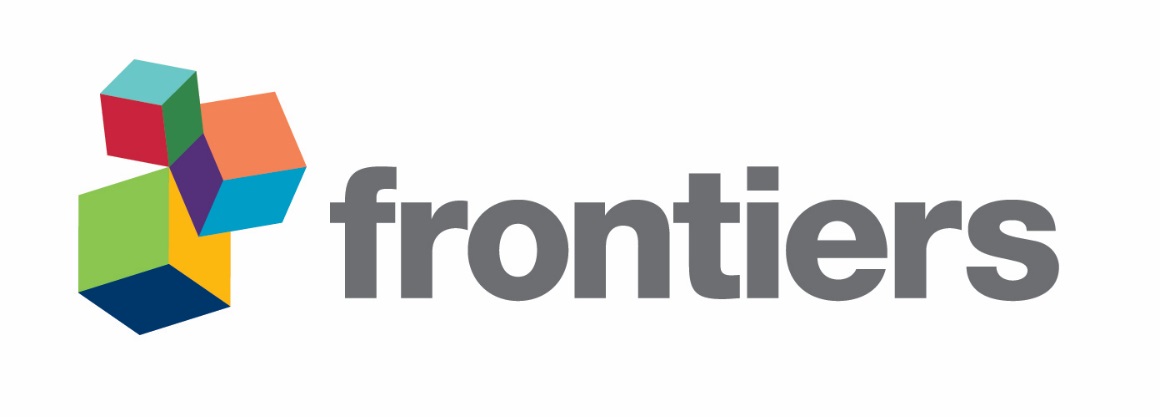
**
